# Association Between Dietary Inflammatory and Oxidative Balance Scores and Skin Cancer Risk: The Mediating Role of Accelerated Phenotypic Aging

**DOI:** 10.3390/cancers18010111

**Published:** 2025-12-29

**Authors:** Shiqi Hui, Zhijia Hou, Dongmei Li

**Affiliations:** 1 Beijing Tongren Eye Center, Beijing Key Laboratory of Intraocular Tumor Diagnosis and Treatment, Beijing Tongren Hospital, Capital Medical University, Beijing 100730, China; 2 Aier Academy of Ophthalmology, Aier Eye Hospital Group Co., Ltd., Changsha 410000, China

**Keywords:** skin cancer, dietary inflammation, dietary oxidation, biological age, NHANES

## Abstract

Background: Skin cancer is known to be associated with aging, oxidative stress, and inflammation. The present study aimed to explore the association between PhenoAge, dietary inflammatory index (DII), and dietary oxidative balance index (DOBS) with skin cancer risk. Methods: A total of 474 individuals aged over 20 years who had information on DII, DOBS, PhenoAge, socioeconomic and demographic factors, and self-reported skin cancer, and 16,154 without skin cancer were included in the National Health and Nutrition Examination Survey database (2005–2018). The combination of DII/DOBS was categorized into 3 categories: inflammation- and oxidation-promoting diet, inflammation- and oxidation-reducing diet, and composite diet. We applied logistic regression to estimate odds ratios (ORs) for the association of DII/DOBS and PhenoAge with skin cancer risk, after adjusting for covariates and survey year. Results: PhenoAge was associated with an increased likelihood of skin cancer (OR 1.07, 95% CI 1.06 to 1.08, *p* < 0.001). DII and DOBS were associated with PhenoAge advancement of OR 1.28 (95% CI 1.20 to 1.36), OR 0.95 (95% CI 0.94 to 0.96), respectively (*p* < 0.001). After adjusting for all covariates, the comparison between the inflammation–oxidation-promoting diet and the inflammation–oxidation-reducing diet had a positive relationship with skin cancer (OR 2.19, 95% CI 1.29 to 3.72, *p* = 0.004). PhenoAge mediated 28.06% of the associations between DII/DOBS and skin cancer risk (*p* < 0.05). The association remained in the subgroup analysis. Conclusion: Our results suggest that an inflammation- and oxidation-promoting diet is related to increased skin cancer risk and may be partly mediated by PhenoAge.

## Simple Summary

Using nationally representative U.S. data (NHANES 2005–2018), we found that individuals consuming an inflammation- and oxidation-promoting diet were more than twice as likely to report skin cancer as those who eat an anti-inflammatory, antioxidant-rich diet. Accelerated biological aging, measured by PhenoAge, explained roughly one-quarter of this diet–cancer link, suggesting that eating more anti-inflammatory and antioxidant foods may slow biological aging and help lower skin-cancer risk.

## 1. Introduction

Skin cancer is one of the most common cancers worldwide and poses a significant threat to global healthcare systems, with incidence rates projected to increase dramatically if not detected early [1,2]. The rising incidence of skin cancer, coupled with its high prevalence, places a significant financial burden on both patients and healthcare systems [3]. While non-melanoma skin cancers contribute relatively low mortality, melanoma represents a leading cause of skin cancer-related deaths, responsible for over 7000 deaths each year. Prevention through daily dietary behaviors is critical to reducing the burden of skin cancer and enabling early prevention, which can mitigate its impact.

Long-term elevated levels of inflammation and oxidative stress have been linked to the risk factors for the onset and progression of various diseases [4,5], as well as accelerated biological aging in conjunction with chronological age. These factors can lead to cellular homeostasis, progressive tissue deterioration, and cumulative organ impairment, thereby contributing to accelerated aging [6]. Epidemiological studies have shown that dietary patterns play a key role in regulating inflammation and maintaining oxidative stress homeostasis [7]. Therefore, it is logical to propose that diet may influence the development of skin cancer through its impact on chronic inflammation and oxidation. Furthermore, diet-induced inflammation- and oxidation-promoting potential might contribute to the connection between accelerated biological aging and the increased risk of skin cancer.

The dietary inflammatory index (DII) and dietary oxidative balance index (DOBS) are dietary assessment tools developed from a summary of published literature, designed to estimate the inflammatory and oxidative properties of nutrients consumed in an individual’s diet, respectively, and to evaluate the influence of dietary patterns on inflammation and oxidative stress [8,9]. Several studies have shown that high DII or low DOBS scores are associated with unfavorable overall health and an increased risk of chronic diseases such as diabetes [10], osteoarthritis [11], cardiovascular disease [12], and cancer [13,14]. While some studies have investigated the relationship between DII and DOBS in the context of cancer, the connection between diet-induced inflammation-oxidation-promoting potential and the risk of skin cancer remains unclear.

Aging is an intricate and unidirectional biological process marked by the progressive deterioration of physiological function and integrity, making it the leading risk factor for cancer [15]. Biological aging refers to the gradual deterioration of biological systems as individuals grow older. A key concept related to biological aging is biological age, which provides an assessment of the aging process across various biological systems [16]. Previous studies suggested that biological aging is modifiable and may offer a more accurate reflection of an individual’s aging status compared to chronological age, as the latter only measures the duration of life lived and does not account for an individual’s health status. Slowing the biological aging process may help reduce the risk or postpone the development of various age-associated disorders [17]. Biological age indicators derived from the relationship between clinical markers and chronological age have been shown to strongly predict mortality, age-associated illnesses, and declines in physical function [18].

However, the mediating effect of dietary inflammation- and oxidation-promoting potential on the relationship between biological aging and skin cancer is not well understood. To test this hypothesis, we examined the mediating role of biological aging in the association of DII/DOBS and skin cancer risk using data from the National Health and Nutrition Examination Survey (NHANES) (2005–2018).

## 2. Materials and Methods

### 2.1. Study Population

**Figure 1:**
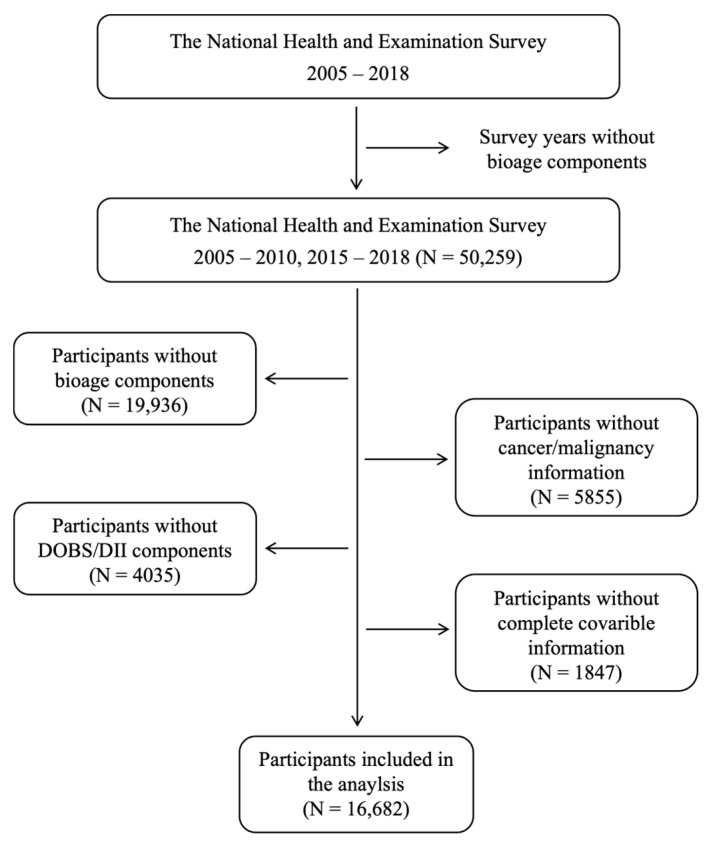
Flow diagram of analytic samples from NHANES 2005–2018.

The data used in this study were sourced from the NHANES of adults (age ≥ 18 years), conducted between 2005 and 2018. NHANES is an ongoing cross-sectional program run by the National Center for Health Statistics at the Centers for Disease Control and Prevention. The survey aims to assess the general health behaviors of the public, as well as their socioeconomic status, nutritional intake, and the results of physical examinations. Individuals were excluded from the study if they met the following criteria: (1) implausible daily energy intake (<800 or >4200 kcal for males, or <500 or >3500 kcal for females) [19], (2) lack of data on DII/DOBS components or covariates, (3) missing physiological characteristics needed to develop biological age indicators, or (4) no outcome data of interest. Ethical approval for the survey was obtained from the Institutional Review Board of NCHS, and all participants were given written informed consent before participation. A flowchart illustrating the participant selection process is shown in Figure 1.

### 2.2. DII Assessment

DII was developed through an extensive literature review and analysis of population data, considering 45 dietary components with the potential to influence inflammation, along with their respective intake levels [8]. Of these 45 components, 26 were selected to calculate the DII. Research has shown that focusing on these specific foods enhances the predictive stability of the DII [20]. Comprehensive details on the dietary elements and calculation methods for the DII are presented in Supplementary Table S1. Each dietary component is calculated using the following formula: (daily intake—global average daily intake)/the standard deviation of global average daily intake × the overall inflammatory effect score of that component. The participant’s total DII is obtained by summing the DII scores of each dietary component. A higher DII score indicates a greater potential for dietary inflammation, and individuals in the highest tertile of DII are categorized as following an inflammation-promoting diet. The DII has been validated in diverse populations and has been consistently associated with chronic inflammation, cardiometabolic diseases, multiple cancer types, and all-cause mortality.

### 2.3. DOBS Assessment

The DOBS was calculated by combining scores from specified oxidation-promoting and reducing components [9]. Based on existing knowledge regarding the associations of certain nutrients with oxidative balance, 17 nutrients were included in the calculation, consisting of 3 pro-oxidants and 14 antioxidants. Numerous studies have demonstrated the importance of these food components in determining DOBS [21,22]. Dietary intake variables were categorized into tertiles, with oxidation-reducing components assigned scores of 1 to 3 in ascending order across these tertiles, while oxidation-promoting components were assigned the opposite. For alcohol intake, participants were classified as non-drinkers (0 g/day), moderate drinkers (0–30 g/day for males and 0–15 g/day for females), and heavy drinkers (more than 30 g/day for males and more than 15 g/day for females), who were assigned scores of 3, 2, and 1, respectively [23]. Additionally, carotene was ln-transformed to achieve a better approximation of normal distribution. Further details regarding the DOBS components are provided in Supplementary Table S2. The overall DOBS for each participant was calculated by summing the scores for 17 established elements. Higher DOBS values reflect greater oxidation-reducing potential in the diet, with individuals in the highest tertile categorized as following an antioxidant-rich dietary pattern.

### 2.4. Diet Category Assessment

To examine the differential effects of inflammation- and oxidation-promoting diet features and inflammation- and oxidation-reducing diet properties, we created a combined indicator using DII and DOBS values [24]. Participants were classified into three categories according to DII and DOBS: an inflammation- and oxidation-promoting diet group (classified in the third tertile of DII and the first tertile of DOBS), an inflammation- and oxidation-reducing diet group (classified in the first tertile of DII and the third tertile of DOBS), and an intermediate group comprising individuals who did not fall into either of the aforementioned categories.

### 2.5. Biological Aging Assessment

Biological age was assessed using clinical laboratory blood chemistry through the “BioAge” R package (version 4.4.2). This package calculates biological aging metrics, KDM, PhenoAge, and HD, derived from the NHANES-III dataset (collected between 1988 and 1994) using elastic-net regression to develop a mortality prediction model. These metrics are individualized measures that integrate data from various clinical biomarkers to assess age-related deficits in system integrity [25]. Biological aging was originally developed and validated as a predictor of all-cause mortality and age-related morbidity, but evidence suggests that accelerated biological aging, as captured by Phenoage, is associated with increased cancer incidence and cancer-related mortality [26]. We calculated biological aging using thirteen relevant variables, including chronological age, albumin (liver), Ln-creatinine (kidney), glucose (metabolic), lymphocyte percentage (immune), mean cell volume (immune), red blood cell distribution width (immune), alkaline phosphatase (liver), white blood cell count (immune), total cholesterol (metabolic), systolic blood pressure, and uric acid (kidney). Although multiple aging indices were calculated, we focused primarily on PhenoAge because it integrates clinical biomarkers reflecting systemic inflammation and metabolic dysregulation, which are mechanistically aligned with DII and DOBS.

### 2.6. Skin Cancer Assessment

Self-reported skin cancer diagnoses were ascertained by the standard question from NHANES: “Have you ever been told by a doctor or other health professional that you had cancer or a malignancy?”

### 2.7. Covariates

Demographic factors, lifestyle behaviors, and characteristics were collected through face-to-face interviews and physical examinations. Demographic information included phenotypic age, gender, race (non-Hispanic White, non-Hispanic Black, Mexican American, and other races), education level (below high school, high school or equivalent, college or equivalent, and above), and household income-to-poverty ratio (PIR). Lifestyle behaviors included tobacco use (measured by cotinine levels in ng/mL) and total energy intake from the 24 h recall (in kcal). BMI was calculated as weight (kg) divided by height squared (m^2^). Self-reported data on hypertension, cardiovascular disease, and diabetes were extracted from the database.

### 2.8. Statistical Analysis

Data from the NHANES 2005–2018 cycles were analyzed. The NHANES employed a complex multi-stage probability sampling method. All analyses were conducted following the complex, stratified design principles outlined in the NHANES analytic and reporting guidelines. Baseline characteristics of the study population were presented as means ± standard deviations for normally distributed continuous variables, medians and interquartile ranges for non-parametric variables, and frequencies with weighted percentages for categorical variables.

Variables with a higher proportion of missing values were classified as unknown, while those with fewer missing values (less than 10%) were imputed using the multiple imputation method provided by the “mi” R package. A difference analysis was performed before and after missing value imputation. Weighted logistic regression was used to estimate odds ratios (ORs) and corresponding 95% confidence intervals (CIs) for the relationship between combined biological aging and skin cancer risks. Weighted multivariable linear regression models were employed to investigate the associations of combined DII/DOBS and PhenoAge. Restricted cubic splines (RCS) models were employed to examine the non-linearity of the association between dietary patterns and PhenoAge. To evaluate whether PhenoAge functions as a mediator in the association between combined DII/DOBS and skin cancer risk, mediation analyses were constructed with adjustments for the above covariates using the “mediation” R package. Additionally, stratified subgroup analyses were carried out to examine the relationship between combined DII/DOBS and skin cancer risk across specific populations. Collinearity analysis was displayed using the “car” R package. A GVIF^(1/(2*Df)) less than 2 was considered to have low collinearity risk.

All analyses were conducted using R software (version 4.4.2; https://www.r-project.org (accessed on 5 April 2025)), with a two-sided *p*-value < 0.05 considered indicative of statistical significance.

## 3. Results

### 3.1. Baseline Characteristics

This analysis included a total of 16,628 U.S. adults. The skin cancer participants had a weighted mean age (±SD) of 68.86 (12.22) years, and 41.8% of them were female. Among participants with skin cancer, individuals tended to be older, predominantly non-Hispanic White, more highly educated, with greater PIR, and exhibited lower serum cotinine concentrations. They also had a higher prevalence of diabetes, hypertension, and cardiovascular disease (*p* < 0.001) (Table 1).

Participants with an inflammation- and oxidation-promoting diet tended to be female, less frequently non-Hispanic White, and more commonly non-Hispanic Black. They were less educated, had lower PIR, higher serum cotinine levels, lower total energy intake, and higher BMI. Additionally, they were more likely to have diabetes, hypertension, and to drink alcohol (*p* < 0.001) (Table 2).

### 3.2. Association Between Aging and Skin Cancer Risks

**Figure 2:**
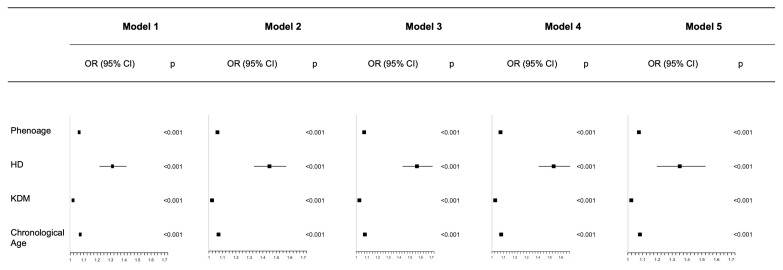
Association between aging and the risk of skin cancer in generalized linear regression models after adjusting for different covariates. Dot points show the estimated values; error bars represent the CI. Model 1: Unadjusted; Model 2: Adjusted for gender and race; Model 3: Further adjusted for education level, PIR, BMI; Model 4: Further adjusted for cotinine, alcohol intake, energy intake; Model 5: Further adjusted for hypertension, diabetes, CHD.

Weighted multivariable logistic regression analyses were performed to examine the relationship between aging and the risk of skin cancer. Both biological aging and chronological aging were strongly linked to a heightened risk of skin cancer across all models. Specifically, PhenoAge (OR 1.074, 95% CI 1.063 to 1.085), HD (OR 1.349, 95% CI 1.196 to 1.522), KDM (OR 1.023, 95% CI 1.017 to 1.028), and chronological age (OR 1.081, 95% CI 1.069 to 1.093) in the fully adjusted Model 5 were all significantly associated with an increased skin cancer risk (*p* < 0.001) (Figure 2). All GVIF^(1/(2*Df) < 1.74.

**Figure 3:**
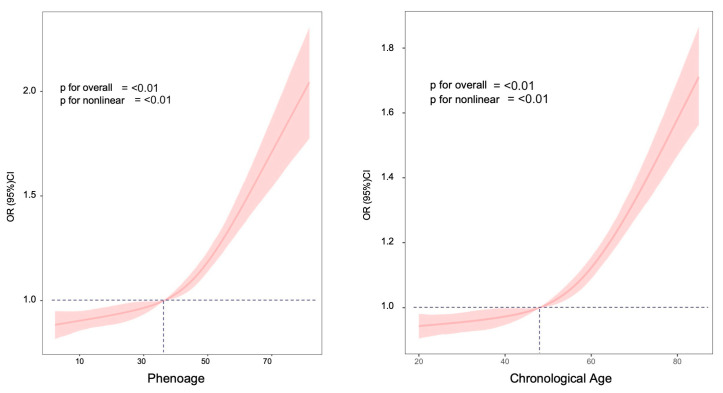
Restricted cubic spline regressions of aging and skin cancer risk. Horizontal dash line represents OR = 1, vertical dash line falls at the corresponding Phenoage/Chronological age. Red shading for CI.

Additionally, we used RCS to examine the dose–response relationship between aging and the risk of skin cancer. After full adjustment, an L-shaped relationship was observed between aging and skin cancer risk, with an inflection point at approximately 36 years old for PhenoAge (non-linear *p* ≤ 0.01 for total, *p* < 0.01 for each DII and DOBS quartile; overall *p* ≤ 0.01 for total, *p* < 0.01 for each DII and DOBS quartile) and at about 48 years old for chronological age (non-linear *p* ≤ 0.01 for total, *p* < 0.01 for each DII and DOBS quartile; overall *p* ≤ 0.01 for total, *p* < 0.01 for each DII and DOBS quartile) (Figure 3 and Supplementary Figure S1).

### 3.3. Association Between Dietary Patterns and Aging

**Figure 4:**
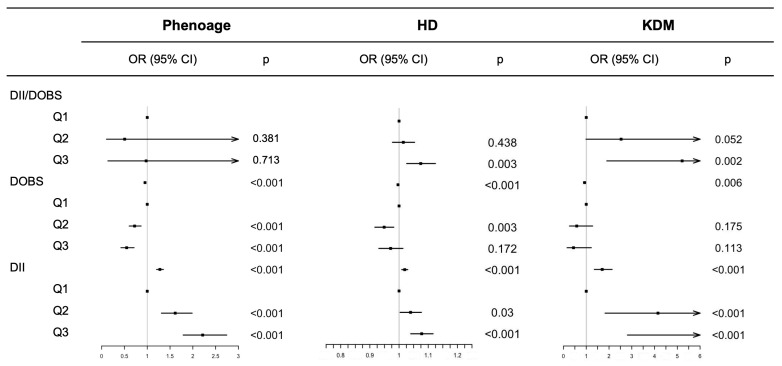
Association of DII, DOBS and different combination of DII/DOBS with aging in generalized linear regression models. Dot points show the estimated values; error bars represent the CI; Arrow for large CI.

Figure 4 and Supplementary Table S3 display the relationship between DII, DOBS, and the combination of DII/DOBS with aging, as assessed by survey-weighted linear regression models. Continued DII was positively associated with increased PhenoAge (β 0.245, 95% CI 0.215 to 0.275), HD (β 0.019, 95% CI 0.014 to 0.024), KDM (β 0.531, 95%CI 0.417 to 0.645), and PhenoAge advance (β 0.245, 95% CI 0.215 to 0.275) (*p* < 0.001). While continues DOBS was negatively associated with increased PhenoAge (β −0.050, 95% CI −0.043 to −0.057), HD (β −0.004, 95% CI −0.003 to −0.005), KDM (β −0.076, 95% CI −0.049 to −0.112), PhenoAge advance (β −0.050, 95% CI −0.043 to −0.057) (*p* < 0.01 or <0.001). An inflammation- and oxidation-promoting diet was significantly associated with increased HD, KDM, and PhenoAge advance (*p* < 0.01 or < 0.001). All GVIF^(1/(2*Df) < 1.96.

### 3.4. Association Between Dietary Patterns and Skin Cancer Risk

**Figure 5:**
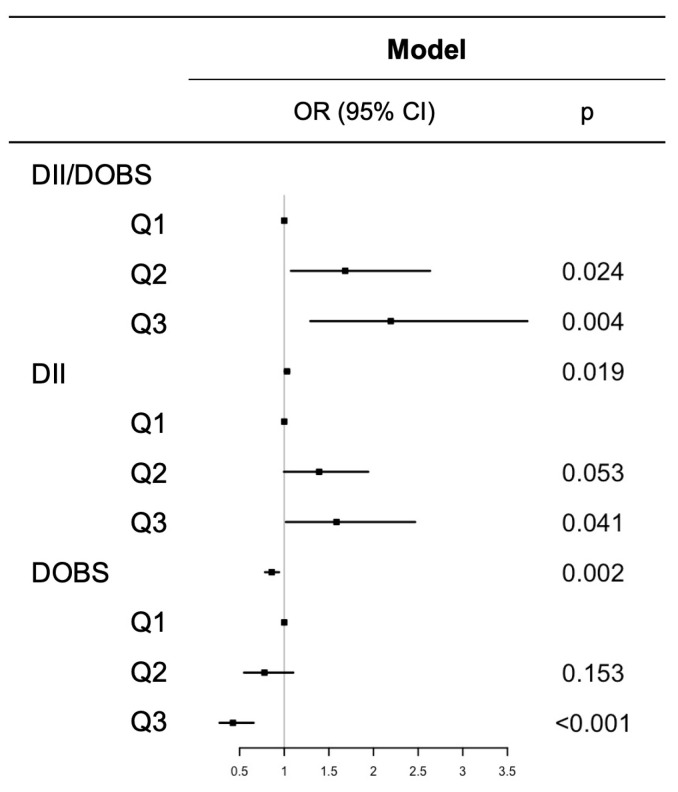
Association of DII, DOBS and different combination of DII/DOBS with the risk of skin cancer in generalized linear regression models. Dot points show the estimated values; error bars represent the CI.

The associations between DII, DOBS, and the combination of DII/DOBS with skin cancer risk are shown by the weighted multivariable logistic regression analyses (Figure 5). After full adjustment, individuals in the highest quartile of DII (OR 1.585, 95% CI 1.019 to 2.466, *p* = 0.041), and DII/DOBS (OR 2.193, 95% CI 1.292 to 3.724, *p* = 0.004) had a higher prevalence of skin cancer compared to those in the lowest quartile. In contrast, individuals in the highest quartile of DOBS (OR 0.425, 95% CI 0.275 to 0.657, *p* < 0.001) were more likely to have skin cancer. All GVIF^(1/(2*Df) < 1.87.

### 3.5. Joint Association Between DII/DOBS, PhenoAge, and Skin Cancer Risk

**Figure 6:**
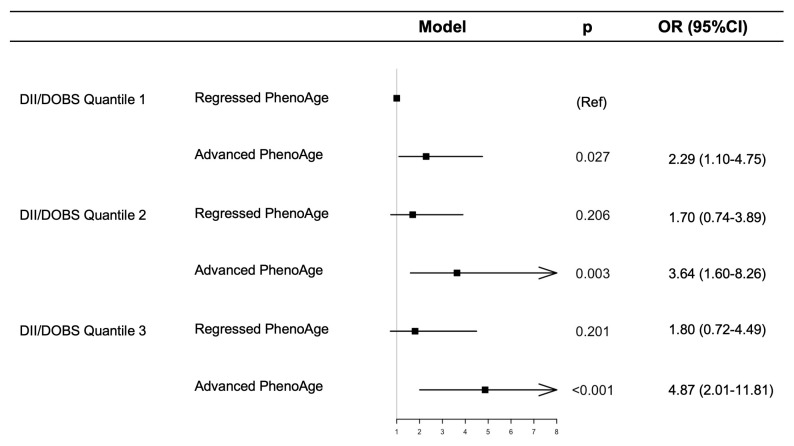
Joint association of DII/DOBS levels and PhenoAge with skin cancer risk among participants. Dot points show the estimated values; error bars represent the CI; Arrow for large CI.

Furthermore, to investigate the relationship between DII/DOBS quantiles, PhenoAge, and skin cancer risk, weighted multivariable logistic regression was performed. Our findings indicated that, compared to the first quartile group (DII/DOBS quantile 1 and regressed PhenoAge), participants in the sixth quartile group (DII/DOBS quantile 3 and advanced PhenoAge) had a 4.87-fold increase in skin cancer risk (95%CI 2.01 to 11.81, *p* < 0.001) after full adjustment (Figure 6). All GVIF^(1/(2*Df) < 1.66.

### 3.6. Mediation Analyses

**Figure 7:**
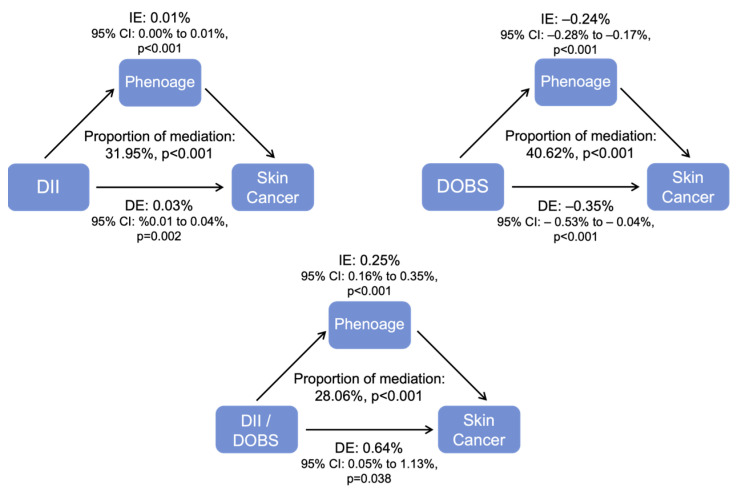
PhenoAge as a mediator in the associations between diet and skin cancer risk.

Mediation effects of biological aging on the relationship of DII, DOBS, and their combined patterns with skin cancer risk were assessed (Figure 7). The indirect effects of PhenAge accounted for 31.95% of the total effect on the relationship of DII with skin cancer risk after adjusting for covariates in the fully adjusted binomial logistic regression model. Regarding DOBS, the indirect effect accounts for 40.62% of the total effect on skin cancer risk. For the DII/DOBS, PhenoAge accounted for 28.06% of the indirect effect, suggesting a partial mediating role of biological aging. This finding implies that while PhenoAge may contribute meaningfully to the observed associations, additional biological, behavioral, and environmental pathways are likely involved.

### 3.7. Subgroup Analyses

Stratified subgroup analyses were performed based on age, gender, race, BMI, PIR, education level, serum cotinine level, alcohol consumption, and the presence of hypertension, CHD, and diabetes (Table 3). The association between DII/DOBS and skin cancer risk was consistent across subgroups. An interaction was observed between race and DII/DOBS in relation to skin cancer risk. A diet with higher DII/DOBS scores (inflammation- and oxidation-promoting diet) was associated with a significantly lower risk of skin cancer in non-Hispanic Black individuals. No significant interactions were found between skin cancer risk and other factors.

## 4. Discussion

We analyzed a nationally representative sample of U.S. adults using the NHANES 2005–2018 cycles. Biological age was assessed using the PhenoAge blood-chemistry algorithm. We examined whether individuals following healthier diets were biologically younger than those with less health-conscious behaviors, and whether those biologically younger were less likely to develop skin cancer. Across all NHANES cycles, a healthier diet and a lower biological age were consistently associated with a reduced likelihood of skin cancer. These associations remained robust after adjusting for potential confounders, including demographic and socioeconomic factors, alcohol intake, smoking, total energy intake, and BMI.

Previous studies have reported that inflammation [27], oxidative stress [28], and aging [29] are among the most significant risk factors for skin cancer. However, few studies have explored the association between diet-derived inflammation and oxidative stress, biological age, and skin cancer. The present study is one of the first to address the lack of data on different dietary patterns and their potential association with biological aging in adults with skin cancer. The sample included adults aged 20 years and older, presenting a diversity of age, race, sex, educational levels, and reported federal poverty levels. The diversity of the sample, along with the nationally representative nature of NHANES data, enhances the generalizability of the findings.

We examined the influences of dietary patterns and biological aging in the U.S. population and whether these influence the possibility of skin cancer. Additionally, we assessed possible compounding adverse effects of having an inflammation- and oxidation-promoting diet and biological aging on skin cancer risk. Our analysis confirmed previous findings on the impact of diet on biological aging [17]. The results revealed statistically significantly higher risk factors in skin cancer individuals with either or both inflammation- and oxidation-promoting dietary patterns and were biologically older. We found that those individuals were 4.87 times more likely to experience skin cancer compared to those with an inflammation- and oxidation-reducing diet and were biologically younger. The higher odds of skin cancer among individuals with co-occurring inflammation- and oxidation-promoting diet and were biologically older confirmed our hypothesis of a possible compounding debilitating impact.

The association between diet behaviors and cancer is increasingly recognized, partly in the context of self-management and individuals’ ability to control their health. Its use in disease management has been shown to confer numerous health benefits. Several studies have shown that certain inflammation- and oxidation-promoting dietary patterns can lead to a range of physical and mental health issues, potentially resulting in metabolic disturbances [30,31]. These findings highlight that an inflammation- and oxidation-promoting diet can negatively affect biological functioning and reduce overall quality of life. They also emphasize the importance of skin cancer survivors considering the impact of their dietary behaviors. Effectively managing dietary habits in patients with skin tumors may enhance their long-term survivorship outcomes.

Biological age has been proven to more accurately reflect an individual’s overall condition, rather than relying solely on chronological age, and provides a more accurate prediction of health-related outcomes [32]. Elevated phenotypic age is strongly associated with age-related comorbidities and declines in physical function, even after adjusting for chronological age. Therefore, incorporating biological age—especially phenotypic age—into clinical assessments may provide a more accurate and comprehensive evaluation of an individual’s health status, enabling more personalized and effective interventions.

Interestingly, we observed a higher proportion of males than females among skin cancer patients, along with lower serum cotinine levels, who tend to be non-Hispanic White, more highly educated, and with higher PIR. Given that smoking is a well-established risk factor for squamous cell carcinoma, its association with other subtypes remains less clear. Female sex is typically linked to a lower risk of squamous cell carcinoma but may be linked to a higher incidence of early-onset basal cell carcinoma and melanoma, findings which may appear counterintuitive. The possible explanation is that individuals with lower socioeconomic status, reflected by lower education levels and PIR, may have limited access to healthcare services and thus are less likely to seek diagnosis or treatment [33]. In contrast, those with higher socioeconomic status may engage more frequently in outdoor recreational activities such as sunbathing, potentially increasing UV exposure [34]. Additionally, variations in skin pigmentation in non-Hispanic Whites could influence susceptibility to sunburn, further modifying skin cancer risk [35].

Several limitations of the present analysis need to be addressed. This analysis was based on data from an observational and cross-sectional dataset. Although mediation analyses were conducted to explore potential pathways, the causal relationships between DII, DOBS, PhenoAge, and skin cancer should be interpreted as hypothesis-generating rather than causal. Reverse causation and residual confounding cannot be fully excluded. Secondly, skin cancer status in NHANES is based on self-reported physician diagnoses rather than systematic clinical verification; this misclassification is likely to be non-differential and may have attenuated the observed associations. Additionally, ultraviolet radiation exposure is a well-established risk factor for skin cancer. However, a detailed measure of lifetime ultraviolet exposure is not consistently available, which may have influenced the observed associations. Dietary intake was calculated from two 24 h dietary recalls, which may not fully reflect long-term habitual dietary patterns, and could therefore misclassify participants with respect to their usual inflammatory or oxidative potential. Furthermore, NHANES does not consistently provide the timing or year of skin cancer diagnosis for all participants, which precludes the determination of the temporal sequence between dietary exposure, biological aging, and skin cancer diagnosis. As such, reverse causation is possible, whereby a prior diagnosis of skin cancer may influence dietary behaviors or aging-related biomarkers. Future longitudinal studies and prospective cohort designs with repeated dietary and aging assessments, as well as more comprehensive covariates, are required to confirm the temporal sequence and causal mechanisms underlying these associations.

## 5. Conclusions

In conclusion, the result of this study highlights the importance of dietary patterns and biological age in individuals with skin cancer. These results provide a foundation for developing hypotheses regarding specific characteristics and underlying mechanisms by which diet influences skin cancer, to be explored in future research. Given that skin cancer can significantly impair both physical and psychological well-being, future research should prioritize strategies to systematically assess and address the multifactorial impacts, including the role of diet and biological aging. Such approaches could lead to more personalized interventions, ultimately improving clinical outcomes and enhancing the quality of life for both patients and survivors.

## Figures and Tables

**Table 1:** Characteristics of participants by skin cancer.

	Total	Non-Skin Cancer	Skin Cancer	*p*
**N**	16,628	16,154	474	
**Gender (Female, %)**	8742 (52.6)	8544 (52.9)	198 (41.8)	**<0.001**
**Age (year, mean (SD))**	49.03 (17.43)	48.44 (17.22)	68.86 (12.22)	**<0.001**
**Age Group (%)**				**<0.001**
≤55	10,363 (62.3)	10,289 (63.7)	74 (15.6)	
55–80	6117 (36.8)	5740 (35.5)	377 (79.5)	
>80	148 (0.9)	125 (0.8)	23 (4.9)	
**Race (%)**				**<0.001**
Mexican American	2876 (17.3)	2867 (17.7)	9 (1.9)	
Other Hispanic	1491 (9.0)	1485 (9.2)	6 (1.3)	
Non-Hispanic White	7603 (45.7)	7154 (44.3)	449 (94.7)	
Non-Hispanic Black	3263 (19.6)	3259 (20.2)	4 (0.8)	
Other Race	1395 (8.4)	1389 (8.6)	6 (1.3)	
**Education Level (%)**				**<0.001**
Under high school	3871 (23.3)	3804 (23.5)	67 (14.1)	
High School Grade or Equivalent	3896 (23.4)	3791 (23.5)	105 (22.2)	
Some College	4954 (29.8)	4806 (29.8)	148 (31.2)	
College Graduate or above	3907 (23.5)	3753 (23.2)	154 (32.5)	
**Family PIR (mean (SD))**	2.60 (1.61)	2.58 (1.61)	3.20 (1.55)	**<0.001**
**Cotinine (ng/mL, mean (SD))**	55.74 (127.10)	56.29 (127.67)	36.98 (104.29)	**<0.001**
**Hypertension (%)**				**<0.001**
No	10,941 (65.8)	10,721 (66.4)	220 (46.4)	
Yes	5687 (34.2)	5433 (33.6)	254 (53.6)	
**Diabetes (%)**				**<0.001**
No	356 (2.1)	342 (2.1)	14 (3.0)	
Broadline	14,222 (85.5)	13,848 (85.7)	374 (78.9)	
Yes	2050 (12.3)	1964 (12.2)	86 (18.1)	
**CHD (%)**				**<0.001**
No	15,955 (96.0)	15,551 (96.3)	404 (85.2)	
Yes	673 (4.0)	603 (3.7)	70 (14.8)	
**Energy intake (kcal/d, mean (SD))**	2000.81 (709.78)	2001.41 (712.32)	1980.44 (617.47)	0.526
**BMI (kg/m^2^, mean (SD))**	29.41 (6.88)	29.44 (6.91)	28.54 (5.86)	**0.005**
**Alchol intake (gm/d, mean (SD))**	7.68 (18.92)	7.66 (18.98)	8.43 (16.87)	0.382

Values marked as bold to indicate statistically significant differences.

**Table 2:** Characteristics of participants according to different diet.

	Anti-Inflammatory Antioxidant Diet	Composite Diet	Proinflammatory Pro-Oxidative Diet	*p*	DOBS T1	DOBS T2	DOBS T3	*p*	DII T1	DII T2	DII T3	*p*
**N**	4263	8069	4296		5400	5674	5554		5568	5554	5506	
**Gender (Female, %)**	1515 (35.5)	4274 (53.0)	2953 (68.7)	**<0.001**	3717 (68.8)	3090 (54.5)	1935 (34.8)	**<0.001**	2303 (41.4)	2904 (52.3)	3535 (64.2)	**<0.001**
**Age (mean (SD))**	48.6 (16.8)	48.7 (17.4)	50.0 (18.1)	**<0.001**	50.5 (18.0)	49.1 (17.5)	47.5 (16.7)	**<0.001**	49.5 (16.8)	48.6 (17.4)	49.0 (18.1)	**0.029**
**Race (%)**				**<0.001**				**<0.001**				**<0.001**
Mexican American	748 (17.5)	1396 (17.3)	732 (17.0)		930 (17.2)	971 (17.1)	975 (17.6)		987 (17.7)	1013 (18.2)	876 (15.9)	
Other Hispanic	357 (8.4)	697 (8.6)	437 (10.2)		539 (10.0)	504 (8.9)	448 (8.1)		476 (8.5)	480 (8.6)	535 (9.7)	
Non-Hispanic White	2174 (51.0)	3728 (46.2)	1701 (39.6)		2146 (39.7)	2629 (46.3)	2828 (50.9)		2738 (49.2)	2518 (45.3)	2347 (42.6)	
Non-Hispanic Black	569 (13.3)	1553 (19.2)	1141 (26.6)		1404 (26.0)	1055 (18.6)	804 (14.5)		778 (14.0)	1107 (19.9)	1378 (25.0)	
Other Race	415 (9.7)	695 (8.6)	285 (6.6)		381 (7.1)	515 (9.1)	499 (9.0)		589 (10.6)	436 (7.9)	370 (6.7)	
**Education Level (%)**				**<0.001**				**<0.001**				**<0.001**
Under high school	691 (16.2)	1856 (23.0)	1324 (30.8)		1617 (29.9)	1276 (22.5)	978 (17.6)		939 (16.9)	1314 (23.7)	1618 (29.4)	
High School Grade or Equivalent	834 (19.6)	1884 (23.3)	1178 (27.4)		1425 (26.4)	1296 (22.8)	1175 (21.2)		1052 (18.9)	1330 (23.9)	1514 (27.5)	
Some College	1250 (29.3)	2472 (30.6)	1232 (28.7)		1533 (28.4)	1750 (30.8)	1671 (30.1)		1633 (29.3)	1690 (30.4)	1631 (29.6)	
College Graduate or above	1488 (34.9)	1857 (23.0)	562 (13.1)		825 (15.3)	1352 (23.8)	1730 (31.1)		1944 (34.9)	1220 (22.0)	743 (13.5)	
**Family PIR (mean (SD))**	3.0 (1.6)	2.6 (1.60)	2.2 (1.5)	**<0.001**	2.2 (1.5)	2.6 (1.6)	2.9 (1.6)	**<0.001**	3.0 (1.6)	2.6 (1.6)	2.2 (1.5)	**<0.001**
**Cotinine (ng/mL, mean (SD))**	36.0 (104.5)	54.4 (124.0)	77.8 (148.1)	**<0.001**	70.8 (142.6)	52.9 (121.7)	44.1 (114.5)	**<0.001**	34.6 (101.8)	53.1 (122.6)	79.8 (148.7)	**<0.001**
**Hypertension (%)**	1321 (31.0)	2740 (34.0)	1626 (37.8)	**<0.001**	2055 (38.1)	1929 (34.0)	1703 (30.7)	**<0.001**	1761 (31.6)	1889 (34.0)	2037 (37.0)	**<0.001**
**Diabetes (%)**				**<0.001**				**<0.001**				**<0.001**
No	97 (2.3)	162 (2.0)	97 (2.3)		126 (2.3)	109 (1.9)	121 (2.2)		132 (2.4)	106 (1.9)	118 (2.1)	
Broadline	3720 (87.3)	6924 (85.8)	3578 (83.3)		4503 (83.4)	4880 (86.0)	4839 (87.1)		4837 (86.9)	4751 (85.5)	4634 (84.2)	
Yes	446 (10.5)	983 (12.2)	621 (14.5)		771 (14.3)	685 (12.1)	594 (10.7)		599 (10.8)	697 (12.5)	754 (13.7)	
**CHD (%)**	166 (3.9)	316 (3.9)	191 (4.4)	0.305	229 (4.2)	237 (4.2)	207 (3.7)	0.327	227 (4.1)	203 (3.7)	243 (4.4)	0.128
**Energy intake (kcal, mean (SD))**	2630.0 (636.9)	2000.7 (560.8)	1376.6 (417.6)	**<0.001**	1402.3 (408.8)	1956.2 (473.5)	2628.3 (610.6)	**<0.001**	2445.4 (684.0)	2021.4 (592.3)	1530.4 (522.6)	**<0.001**
**BMI (kg/m^2^, mean (SD))**	28.7 (6.4)	29.5 (6.9)	30.0 (7.4)	**<0.001**	29.8 (7.2)	29.3 (6.8)	29.1 (6.6)	**<0.001**	28.6 (6.3)	29.6 (6.9)	30.1 (7.4)	**<0.001**
**Alcohol intake (gm, mean (SD))**	9.2 (19.3)	8.2 (20.2)	5.2 (15.6)	**<0.001**	6.5 (17.5)	8.3 (20.5)	8.2 (18.5)	**<0.001**	9.7 (19.8)	8.4 (20.8)	4.9 (15.3)	**<0.001**

Values marked as bold to indicate statistically significant differences.

**Table 3:** Stratified analyses of associations between DII/DOBS and skin cancer risk among participants.

Characteristic	OR (95%CI)	*p*	*p* for Interaction
**Gender**			0.812
Male	1.36 (1.00, 1.86)	0.054	
Female	1.15 (0.80, 1.66)	0.445	
**Age**			0.135
≤55	1.05 (0.68, 1.62)	0.820	
55–80	1.27 (0.93, 1.74)	0.149	
>80	8.82 (2.22, 35.03)	**0.005**	
**Race**			**0.018**
Mexican American	1.59 (0.71, 3.56)	0.271	
Other Hispanic	0.73 (0.17, 3.23)	0.682	
Non-Hispanic White	1.34 (1.05, 1.71)	**0.026**	
Non-Hispanic Black	0.01 (0.00, 0.23)	**0.007**	
Other Race—Including Multi-Racial	0.06 (0.00, 1.27)	0.082	
**Education level**			0.051
Under high school	2.44 (1.32, 4.50)	**0.007**	
High School Grade or Equivalent	1.44 (0.97, 2.15)	0.079	
Some College	1.18 (0.74, 1.88)	0.494	
College Graduate or above	0.98 (0.64, 1.52)	0.939	
**BMI (kg/m^2^)**			0.616
≤18.50	0.59 (0.04, 8.99)	0.709	
18.51–24.00	1.44 (0.83, 2.51)	0.205	
>24.00	1.20 (0.91, 1.57)	0.197	
**PIR (%)**			0.148
≤1	2.05 (0.89, 4.75)	0.098	
>1	1.23 (0.95, 1.59)	0.125	
**Serum Cotinine (ng/mL)**			0.558
≤0.019	0.75 (0.56, 1.00)	0.055	
0.020–0.234	0.92 (0.55, 1.53)	0.741	
>0.234			0.324
**Alcohol (g/d)**	1.25 (0.89, 1.74)	0.200	
≤15	1.14 (0.61, 2.12)	0.688	
15–30	1.35 (0.79, 2.30)	0.283	
>30			0.727
**Diabetes**	1.23 (0.94, 1.60)	0.137	
No	1.75 (0.73, 4.16)	0.216	
Broadline	1.22 (0.59, 2.49)	0.595	
Yes			0.39
**CHD**	9.99 (1.86, 53.76)	**0.011**	
No	1.16 (0.88, 1.54)	0.305	
Yes	1.80 (0.98, 3.33)	0.066	
**Hypertension**			0.21
No	1.31 (0.99, 1.74)	0.062	
Yes	0.92 (0.56, 1.54)	0.762	
**Hypertension**			0.189
No	1.02 (0.72, 1.46)	0.896	
Yes	1.48 (1.01, 2.17)	**0.049**	

Values marked as bold to indicate statistically significant differences.

## Data Availability

The data in this study are publicly available and can be found on the corresponding website. R 4.4.2 scripts enabling the main steps of the analysis are not publicly available, but may be available from the corresponding author on reasonable request.

## Supplementary Materials

The following supporting information can be downloaded at: https://www.mdpi.com/article/10.3390/cancers18010111/s1, Figure S1: Restricted cubic spline regressions of aging and skin cancer risk stratified by different DII and DOBS. Table S1: Dietary composition used for DII construction. Table S2: Dietary composition used for DOBS construction. Table S3: Association of dietery patterns with aging.
