# Supplementary material for: Association Between Dietary Inflammatory and Oxidative Balance Scores and Skin Cancer Risk: The Mediating Role of Accelerated Phenotypic Aging
Source: Cancers (Basel). 2025 Dec 29;18(1):111. doi: 10.3390/cancers18010111 (PMC12784734; doi:10.3390/cancers18010111)
Supplement: Supplementary file 1 [file cancers-18-00111-s001.zip › cancers-4047205-supplementary.pdf]

Figure S1. Restricted cubic spline regressions of aging and skin cancer risk stratified by different DII and DOBS.

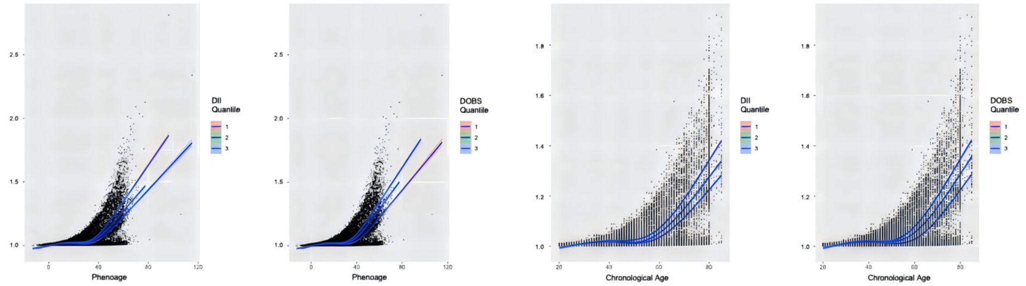

Table S1. Dietary composition used for DII construction.

Dietary composition	Raw inflammatory effect score	Overall inflammatory effect score	Global daily mean intake (units/d)	Standard deviation of the global daily intake
Alcohol (g)	-0.28	-0.28	13.98	3.72
Vitamin B12 (μg)	0.21	0.11	5.15	2.70
Vitamin B6 (mg)	-0.38	-0.37	1.47	0.74
β-Carotene (μg)	-0.58	-0.58	3718.00	1720.00
Caffeine (g)	-0.12	-0.11	8.05	6.67
Carbohydrate (g)	0.11	0.10	272.20	40.00
Cholesterol (mg)	0.35	0.11	279.40	51.20
Energy (kcal)	0.18	0.18	2056.00	338.00
Total fat (g)	0.30	0.30	71.40	19.40
Fiber (g)	-0.66	-0.66	18.80	4.90
Folic acid (μg)	-0.21	-0.19	273.00	70.70
Iron (mg)	0.03	0.03	13.35	3.71
Magnesium (mg)	-0.48	-0.48	310.10	139.40
MUFA (g)	-0.02	-0.01	27.00	6.10
Niacin (mg)	-1.00	-0.25	25.90	11.77
Protein (g)	0.05	0.02	79.40	13.90
PUFA (g)	-0.34	-0.34	13.88	3.76
Vitamin B2 (mg)	-0.73	-0.07	1.70	0.79
Saturated fat (g)	0.43	0.37	28.60	8.00

Selenium (μg)	-0.19	-0.19	67.00	25.10
Vitamin B1 (mg)	-0.35	-0.10	1.70	0.66
Vitamin A (RE)	-0.40	-0.40	983.90	518.60
Vitamin C (mg)	-0.42	-0.42	118.20	43.46
Vitamin D (μg)	-0.45	-0.45	6.26	2.21
Vitamin E (mg)	-0.42	-0.42	8.73	1.49
Zinc (mg)	-0.31	-0.31	9.84	2.19

DII of a certain dietary component = (Daily intake of the dietary component - Global daily mean intake of the dietary component) / Standard deviation of the global daily intake for the dietary component * Overall inflammatory effect score of the dietary component. The DII for each participant was obtained by summing the DII of the 26 dietary components selected in this study.

Table S2. Dietary composition used for DOBS construction.

Dietary composition	Property	DOBS score		
		1	2	3
Dietary fiber (g/d)	Antioxidant	≤ 11.95	11.96 - 18.35	＞18.35
Ln-transformed carotene (μg/d)	Antioxidant	≤ 6.39	6.40- 7.59	＞7.59
Vitamin B2 (mg/d)	Antioxidant	≤ 1.52	1.53 - 2.22	＞2.22
Niacin (mg/d)	Antioxidant	≤ 18.33	18.34 - 26.73	＞26.73
Vitamin B6 (mg/d)	Antioxidant	≤ 1.44	1.45 - 2.15	＞2.15
Folic acid (μg/d)	Antioxidant	≤ 100.50	100.51 - 190.50	＞190.50
Vitamin B12 (μg/d)	Antioxidant	≤ 2.95	2.96 - 5.26	＞5.26
Vitamin C (mg/d)	Antioxidant	≤ 41.30	41.31 - 95.55	＞95.55
Vitamin E (mg/d)	Antioxidant	≤ 5.22	5.23 - 8.19	＞8.19
Calcium (mg/d)	Antioxidant	≤ 656.00	656.01 - 1000.50	＞1000.50
Magnesium (mg/d)	Antioxidant	≤ 224.5	224.51 - 316.50	＞316.50
Zinc (mg/d)	Antioxidant	≤ 8.09	8.10 - 12.14	＞12.14
Copper (mg/d)	Antioxidant	≤ 0.93	0.94 - 1.33	＞1.33
Selenium (μg/d)	Antioxidant	≤ 83.35	83.36 - 121.10	＞121.10
Total fat (g/d)	Pro-oxidant	＞85.77	57.39 - 85.77	≤ 57.38
Iron (mg/d)	Pro-oxidant	＞15.94	10.82 - 15.94	≤ 10.81
Alcohol (male) (g/d)	Pro-oxidant	＞30.01	0.01 - 30.00	0.00
Alcohol (female) (g/d)	Pro-oxidant	＞15.01	0.01 - 15.00	0.00

Table S3. Association of dietery patterns with aging.

	PhenoAge		HD		KDM		Chronological Age		PhenoAge Advance	
	OR (95% CI)	P	OR (95% CI)	P	OR (95% CI)	P	OR (95% CI)	P	OR (95% CI)	P
**Inflammation- and Oxidation- Reducing Diet**	(Ref)		(Ref)		(Ref)		(Ref)		(Ref)	
**Composite Diet**	0.505 (0.106, 4.777)	0.381	1.015 (0.977, 1.053)	0.438	2.532 (0.990, 6.474)	0.052	0.084 (0.030, 0.234)	**<0.001**	1.657 (1.341, 2.047)	**<0.001**
**Inflammation- and Oxidation- Promoting Diet**	0.976 (0.137, 7.109)	0.713	1.074 (1.026, 1.125)	**0.003**	5.213 (1.902, 14.290)	**0.002**	0.008 (0.002, 0.030)	**<0.001**	2.654 (2.016, 3.494)	**<0.001**
**DOBS Quartile 1**	(Ref)		(Ref)		(Ref)		(Ref)		(Ref)	
**DOBS Quartile 2**	0.723 (0.604, 0.865)	**<0.001**	0.949 (0.917, 0.982)	**0.003**	0.582 (0.265, 1.280)	0.175	2.646 (1.153, 6.069)	**0.022**	0.723 (0.604, 0.865)	**<0.001**
**DOBS Quartile 3**	0.548 (0.423, 0.711)	**<0.001**	0.971 (0.931, 1.013)	0.172	0.440 (0.158, 1.221)	0.113	13.702 (3.795, 49.474)	**<0.001**	0.548 (0.423, 0.711)	**<0.001**
**DOBS**	0.951 (0.938, 0.964)	**<0.001**	0.996 (0.994, 0.998)	**<0.001**	0.927 (0.879, 0.977)	**0.006**	1.198 (1.113, 1.290)	**<0.001**	0.951 (0.938, 0.964)	**<0.001**
**DII Quartile 1**	(Ref)		(Ref)		(Ref)		(Ref)		(Ref)	
**DII Quartile 2**	1.613 (1.311, 1.986)	**<0.001**	1.039 (1.004, 1.076)	**0.030**	4.150 (1.827, 9.429)	**<0.001**	0.101 (0.040, 0.254)	**<0.001**	1.613 (1.311, 1.986)	**<0.001**
**DII Quartile 3**	2.216 (1.788, 2.745)	**<0.001**	1.078 (1.040, 1.117)	**<0.001**	6.337 (2.810, 14.291)	**<0.001**	0.008 (0.003, 0.022)	**<0.001**	2.216 (1.788, 2.745)	**<0.001**
**DII**	1.278 (1.204, 1.355)	**<0.001**	1.019 (1.009, 1.030)	**<0.001**	1.701 (1.354, 2.136)	**<0.001**	0.276 (0.202, 0.376)	**<0.001**	1.278 (1.204, 1.355)	**<0.001**

Values marked as bold to indicate statistically significant differences.
